# Corporate Volunteering: Relationship to Job Resources and Work Engagement

**DOI:** 10.3389/fpsyg.2018.01884

**Published:** 2018-10-04

**Authors:** Eva Boštjančič, Sandra Antolović, Vanja Erčulj

**Affiliations:** ^1^Department of Psychology, Faculty of Arts, University of Ljubljana, Ljubljana, Slovenia; ^2^Faculty of Criminal Justice and Security, University of Maribor, Maribor, Slovenia

**Keywords:** employee volunteering, corporate volunteering climate, job resources, work engagement, social responsibility

## Abstract

Employers are increasingly including volunteer activities in their social responsibility programs. At companies at which this is done in a planned manner, we can speak of the development of a corporate volunteering, which correlates with numerous positive psychological outcomes at both the individual and the organizational level. The aim of the study was to investigate the relationship between the corporate volunteering programs and job characteristics, connected with work engagement. In our study we were interested in identifying the role of the corporate volunteering in the evaluation of job resources and work engagement. The study included 274 employees from 15 Slovenian companies, of whom 62% participate in their organizations’ volunteer activities. They filled out the Job demands and resources questionnaire, the Utrecht Work Engagement Scale (UWES-17) and a scale for measuring the corporate volunteering climate. The results indicate that the carrying out of volunteer activities correlates with the perception of the corporate volunteering climate. Employees whose employers implement volunteering programs are more engaged and report higher levels of both autonomy and support from their co-workers and supervisors. Theoretical and practical implications are discussed.

## Introduction

Today, volunteers play an important role in addressing numerous social problems, from poverty and hunger to assisting victims of natural disasters, and political issues, such as migrants in recent times. Approximately 140 million people around the world engage in volunteering and thereby contribute 400 billion US dollars to the global economy annually (Wu, 2011). Volunteering is done in employees’ free time, but recently it has also been introduced at work, with some employers including volunteer activities as part of their social responsibility programs. After the end of the global economic crisis in 2015 (Kohont and Stanojević, 2017), the labor market has also changed considerably (Mucci et al., 2016) due to different social and economic changes (Giorgi et al., 2015). The economic capacity and consequent power of organizations have increased and made a place for various benefit programs devoted to broader society also. At that step it is important to identify various advantages and disadvantages of the organizations’ form and how it is utilized in practice. Corporate volunteering has attracted the growing interest of researchers, particularly in the area of organizational psychology.

The advantages that both employers and employees can gain from volunteering motivated us to research the role of the corporate volunteering. The aim of this study was to investigate the relationship between the corporate volunteering programs and job characteristics, as a potential source of advocacy for voluntarism, and with work engagement, a construct that has also played a significant role in the organizational environment recently. Thus our research can contribute to encouraging companies to introduce their own volunteer programs as a form of corporate social responsibility.

### Definition of Corporate Volunteering

The Joint Report on Volunteering in Slovenia (Ministry of Public Administration, 2017) indicates that the number of volunteers is growing, from 96.822 in 2015 to 307.262 in 2016, which is associated with a large number of volunteer organizations were entered in the companies register in 2016. Although there is no data available for Slovenia regarding how many of these individuals are employed full-time, the available sources indicate that encouraging employees to engage in volunteer work is a form of corporate social responsibility that has appeared within organizations throughout the world in recent years. Today, both the corporate world and society as a whole are promoting the notion that employers are not only responsible for creating jobs and profits, but should also be thinking about how their organizations affect society and the general public. Many companies have already introduced corporate volunteering programs, and others are planning to do so (Rodell et al., 2017), which has led to a growing need for research in this area (Rodell et al., 2017).

After addressing and discussing various aspects of voluntarism, the majority of researchers had accepted the behavioral definition of corporate volunteering as “giving time or skills during a planned activity for a volunteer group or organization (e.g., a charity or a non-profit organization)” (Rodell, 2013, p. 1274). Rodell (2013) defines three significant components of voluntarism. The first is donating time instead of just money, which is a passive form of support (Wilson, 2000) – some volunteers assist by donating their skills and professional knowledge, while others do things that are outside of their professional fields (Rodell et al., 2016). The second is that volunteering is a planned activity that requires fast decision-making with regard to the measures taken (Wilson, 2000). Thus e.g., employees who sign up for volunteer work at homes for the elderly are volunteers, while one who 1 day spontaneously decides to help by delivering food to an elderly woman is not. The third component is the volunteer organizations in which the culture of voluntarism develops. This is a formalized and public activity in which the volunteers usually do not know the recipients of their help beforehand (Wilson, 2000; Rodell, 2013; Rodell et al., 2016).

There are several viewpoints regarding corporate volunteering. Some authors (e.g., Wilson, 2000), who define it in terms of its social aspect, believe that employees decide to engage in volunteering voluntarily, i.e., they are not instructed to do so. Other authors avoid including motives when defining voluntarism, particularly in the context of corporate volunteering, as the debates about this are still ongoing and thus make it more difficult to form an operational construct (cf. Rodell, 2013; Rodell et al., 2016; Breitsohl and Ehrig, 2016). Motivations for volunteering range from the personification of one’s own values by socializing with others to escaping from one’s personal problems. Other motives appear in corporate volunteering, such as recognition of supervisors, making an impression on supervisors or co-workers, or improving one’s career opportunities (Booth et al., 2009). Time is also one of the factors that affect defining corporate volunteering – some authors define it as an activity that full-time employees engage in only in their spare time, others as an activity that they participate in due to the initiative of their employer, while still others include both options in their research (Booth et al., 2009; Rodell, 2013). One question that remains open to debate is to what extent the individuals who participate in their employers’ volunteer programs are in fact volunteers if they volunteer only during work time and are therefore paid for their work (Rodell et al., 2016). In view of the fact that the concept of corporate volunteering is still relatively unknown in Slovenia, in our research we will define corporate volunteering as an activity in which employees participate both during their free time and their work hours.

Organizations that engage in volunteering implement “formal and informal practices and policies in order to support their employees and allow them to spend time doing volunteer activities” (Rodell et al., 2017). The most common practices are time-related benefits such as flexible work time and paid leave, financial benefits such as reimbursement of costs, various gifts, event tickets, purchase of the necessary materials, and logistical support, such as use of the employer’s buildings and equipment, and transport at company expense (Booth et al., 2009; Rodell et al., 2017). Employers can enter into pre-planned formal agreements with volunteer organizations, or more informal and flexible arrangements. In formal agreements, companies cooperate with specific volunteer organizations and create volunteer programs that correspond with or are part of their business vision. In informal arrangements, employees have greater autonomy in choosing what volunteer organization they are going to join, and there are fewer rules and administrative formalities (Booth et al., 2009).

Corporate volunteering may develop and spread quickly, since it can be considered a form of corporate social responsibility (Grant, 2012) – “companies can become socially responsible by integrating social, environmental, ethical, consumer and human rights concerns into their business strategy and operations” (European Commission, 2018). It has been shown that employees value volunteering as a form of corporate social responsibility more highly than other forms of philanthropic contributions, specifically due to the more personal engagement with the community at large (JA WorldWide, 2009).

### Corporate Volunteering Climate

The corporate volunteering climate (Rodell et al., 2017) is a perception or belief shared by employees with regard to the employees’ participation in their employer’s volunteer program. This climate reflects a sense within the organization that volunteering behavior is “something that people do here” (Rodell et al., 2017).

The results of a study by Rodell et al. (2017), in which they used a modified version of the scale for measuring volunteering (Rodell, 2013) to measure the corporate volunteering climate, indicated the appearance of a corporate volunteering climate at a company is the result of both a bottom-up and a top–down process, which can complement each other. This climate is created by both employees who express interest in and are passionate about volunteering, and by the employer through the development and implementation of volunteer programs. And when a corporate volunteering climate is created, individual employees don’t have to participate in the volunteer programs to perceive it. In our research we were therefore interested in how the corporate volunteering climate is perceived at companies both by those who actively participate in them and by those who do not.

Hypothesis 1: Employee participation in corporate volunteering increases the perception of the corporate volunteering climate.

### Job Demands-Resources Model

Numerous studies indicate that job characteristics have a major influence on employees’ wellbeing, i.e., they can be indicators of burnout or engagement with the job, and therefore also of the success of the organization. The model that describes this relationship is the Job Demands-Resources (JD-R) model (Demerouti et al., 2001; Bakker and Demerouti, 2007, 2017), which is based on the assumption that every job has certain risk factors associated with it, which can be classified as job demands and resources (Bakker and Demerouti, 2007).

Job demands refer to the physical, psychological, social, and organizational aspects of work that demand sustained physical and/or psychological effort and that can eventually lead to physiological and/or psychological damage. Job demands are not necessarily negative, but they can become job stressors if they exceed the employee’s ability to adapt to them. Examples of job demands include high pressure at work, an unhealthy physical environment, and heavy workloads or difficult interactions with customers. If they are too high, it can lead to exhaustion and medical issues (Bakker et al., 2003; Bakker and Demerouti, 2007).

Job resources are the physical, psychological, social, and organizational aspects of work that promote the achieving of targets and reduce job demands, and therefore physiological or psychological damage, and promote personal growth, learning and development (Bakker et al., 2003; Bakker and Demerouti, 2007). Resources at the organizational level include wages, career opportunities and job safety; at the interpersonal level they include support from co-workers, supervisors and the work environment; at the work organization level they include clearly defined roles and participation in decision-making; and at the level of job duties they include feedback, autonomy and the importance of one’s job (Bakker et al., 2003). A lack of job resources makes it impossible to reach targets, which can lead to failure and frustration, which can consequently lead to decreased motivation and absenteeism. If employees do not have sufficient job resources in their work environment, they are unable to reduce the potential negative influences of a high level of job demands and are thus unable to reach the set targets (Bakker et al., 2003; Bakker and Demerouti, 2007).

The JD-R model assumes that job resources have motivational potential and lead to high levels of engagement and dedication to the organization, and consequently high rates of success. They can play either an internal motivational role, as they promote employee growth, learning and development, or an external motivational role, since they are crucial to the achieving of targets. Therefore work environments that have an abundance of job resources encourage employees to focus their efforts and their skills on their work duties (Bakker and Demerouti, 2007), as well as on volunteer activities that are carried out as part of their regular jobs.

Hypothesis 2: Job resources are positively correlated with the corporate volunteering.

### Factors in the Work Environment Have an Influence on Employee Engagement

Employee engagement is increasingly attracting the interest of both researchers and practitioners, as it significantly contributes to various positive individual and organizational outcomes. This is no surprise, since in recent years an increasing number of companies are looking for employees who are proactive, energetic and dedicated self-starters. In order for modern organizations to compete effectively, they have to have employees who are not just talented in their fields, but are also psychologically connected to their work and fully committed to their work and to achieving high standards of quality. The most sought-after employees are those who are engaged with their work (Bakker and Leiter, 2010).

Employee engagement is most often defined as a positive, fulfilling work-oriented state of mind, which is conditioned by vigor, dedication and absorption (Schaufeli et al., 2002). The vigor component describes work as stimulating and energy-giving, something that employees enjoy spending their time and effort on, the dedication component describes it as something important and meaningful, and the absorption component as something that employees focus their interest and attention on (Bakker et al., 2008). Work engagement is in fact a specific construct, different from constructs such as loyalty to the organization or work inclusion, with which it is occasionally conflated in practice (Bakker and Leiter, 2010). It is positively associated with constructs such as work performance, productivity (Harter et al., 2013), organizational commitment (Kim et al., 2017), organizational identification, job satisfaction (Karanika-Murray et al., 2014).

According to Saks (2006), the degree of employee engagement varies in response to the amount of resources (i.e., performance feedback, social support, and supervisory coaching; Schaufeli and Bakker, 2004) provided by the organization. In addition, studies have shown weak positive correlations between employee engagement and job demands that are stressful but at the same time foster employees’ curiosity, competence, and thoroughness (i.e., job challenges such as responsibility, workload, and cognitive demands) (Crawford et al., 2010). Research findings have also indicated that job resources are most beneficial and may become more salient for work engagement when job demands are high (Hakanen et al., 2005; Bakker et al., 2007). Altogether, challenging jobs characterized by abundant job resources were shown to promote work engagement.

At the individual level, employee self-evaluations play an important role, as they are associated with the individual’s sense of control and effective influence on his environment (e.g., optimism, self-initiative, self-respect) (Crawford et al., 2010) and indicate the degree of work motivation, the achieving of set targets, ambitions, success and work and life satisfaction. People with large amounts of personal resources (i.e., “psychological capital”) have more internal motivation to reach their personal goals and consequently are more successful and satisfied (Bakker and Demerouti, 2008). Xanthopoulou et al. (2007) found that in addition to job resources, personal resources such as self-initiative, organizational self-image and optimism contribute to explaining the variances in employee engagement over time. Engaged individuals are energetic and feel connected to their work, and see themselves as capable of handling their workloads (Schaufeli and Bakker, 2003, Unpublished). They have high levels of energy and self-efficacy and a positive attitude toward their work, and are very active in it (Bakker et al., 2011). They experience positive emotions more often, which might correlate with their productivity – happy people are more open to new opportunities at work, help their co-workers more, are self-confident and optimistic, and as such are also better able to increase their psychological capital (Bakker and Demerouti, 2008). They are capable of creating their own resources, which might also correlate with their higher rates of success in comparison with non-engaged individuals. Job and personal resources promote employee engagement, and at the same time engaged people generate more resources, which further promotes their continued engagement.

Job resources affect people’s engagement in the future, and trigger a motivational process through which employees satisfy their personal needs, such as the need for autonomy, competence and relatedness (Bakker and Leiter, 2010). It has been shown that engaged employees spread their optimism, positive viewpoints and proactive behavior to their co-workers, create a positive climate and thus also affect their work, owing to which the entire team is more successful (Bakker and Demerouti, 2008). Due to all of the positive impacts that job resources have on employees, working teams and the organization as a whole, below we investigate how the corporate volunteering could affect the development of employee engagement.

### The Effects of Corporate Volunteering at the Employee and Organizational Level

Past studies (e.g., Grant, 2012; Rodell, 2013) have researched what job characteristics are associated with employee volunteering and/or what effects they have on it. It turned out that individuals who see their jobs as important and meaningful – they do work that has a significant and lasting effect on others, see the big picture, i.e., finish entire projects from start to finish, have autonomy in decision-making and receive positive feedback on their performance (Grant, 2012) – join their employers’ volunteer programs due to a sense of belonging and as an expression of gratitude. In contrast, those who do not have a sense that their work is meaningful and do not see it as important try to compensate for these deficiencies through engaging in volunteer activities (Grant, 2012; Rodell, 2013). Employees choose to participate in corporate volunteering activities for a number of reasons, e.g., because they are directly asked to, due to feeling pressured by co-workers or supervisors, or due to loyalty to the organization, or because of paid vacations, various incentives, donations and other benefits that increase the desirability of participating in their organization’s volunteer programs (Grant, 2012). However, due to their daily close interactions and their effects on people’s emotions and experiences at work, supervisors and co-workers can also represent social models that motivate employees to participate in volunteer activities (Hu et al., 2016), rather than solely being agents of social pressure.

Previous research (e.g., Rodell et al., 2016) has shown that employees’ participation in their employers’ volunteer programs has positive outcomes at both the individual (personal outcomes) and organizational levels (job performance, external recognition of the organization, etc.). Corporate volunteering allows employees to connect with others and experience a sense of belonging and purpose in life (Rodell, 2013; Brockner et al., 2014), and they report having experiences that allowed them to grow and develop (Booth et al., 2009). Voluntarism is also associated with employees’ wellbeing through the satisfaction of psychological needs; it has been shown that participating in volunteer activities improves one’s emotional state and correlates positively with the expression of more positive and fewer negative emotions at the workplace (Mojza et al., 2011).

The effects of volunteering are also felt at the organizational level (cf. Harter et al., 2013; Karanika-Murray et al., 2014; Kim et al., 2017), since employed volunteers show better performance results and remain at their companies for longer periods of time (Rodell et al., 2016), feel a higher sense of belonging to the organization (Breitsohl and Ehrig, 2016), are more loyal to the organization and have higher levels of job satisfaction (Caudron, 1994). In addition, they identify with their companies more (Rodell et al., 2017) and possess better work-related skills such as communication, interpersonal skills, dedication, creativity and active listening.

Employees nowadays often expect their employers to express concern for the community at large and consequently to include volunteer programs as part of their regular activities. It was shown that employees who participate in volunteer programs and thereby satisfy their own personal needs are also more engaged in their work (Allen, 2013). However, there is relatively little other evidence of the connection between employee engagement and corporate volunteering.

Corporate volunteering has positive effects not only on behavior within the organization, but also on its image among members of the public such as customers, potential job candidates or investors. Studies have shown that companies that offer or run volunteer programs are more attractive to job seekers, particularly for members of Generation Y, who are able to recognize socially responsible employers on the job market (Deloitte, 2011; Rodell et al., 2016).

Hypothesis 3: Corporate volunteering positively correlates with employee engagement.

## Materials and Methods

### Participants

The questionnaire was sent to every employee in 44 organizations in various professional fields. A total of 347 employees answered the questionnaire, resulting in a response rate of 16.2%. Complete data were available for 318 employees working at 43 organizations. The minimum number of respondents per organization was one and the maximum was 78 respondents. As the respondents were nested within the organizations, mixed linear regression models were used to test the hypotheses. Due to sample size requirements on both levels of the analysis, only organizations with at least 8 participating employees were included in the analysis. The organizations analyzed (*N* = 15) were mostly Slovenian-owned (71.4%), and came from various fields (processing activities, education, information and communications, supply of electricity, gas and steam, arts and culture, entertainment and recreation, energy, public administration and defense).

The final sample size therefore consisted of 274 respondents working at 15 companies. The mean age of the respondents was 40.1 years (*SD* = 9.8). The sample included 156 (56.9%) women and 143 (52.2%) respondents with at least a Master’s degree. 170 (62%) of the employees are employed in companies that implement corporate volunteering programs.

### Measures

#### Job Characteristics

In line with the JD-R model (Bakker and Demerouti, 2007), Job demands and resources questionnaire – JDRQ (Tement et al., 2010) is based on Perceived work demand scale (PWD; Boyar et al., 2007) and Job content questionnaire (JCQ; Karasek et al., 1998). Authors report no validation results (Tement et al., 2010). JDRQ includes five different job characteristics measured using 18 items on a five-point Likert scale ranging from 1 (strongly disagree) to 5 (strongly agree). Work variety was measured using two items. Ex.: “My job requires learning new things.” Cronbach’s alpha in this section was 0.73. Perceived workload was measured using five items. Ex.: “I feel like I have a lot of job demands.” Cronbach’s α was 0.88. Autonomy, the degree of decision-making freedom at work, was measured using three items. Ex.: “My job allows me to make decisions on my own.” Cronbach’s alpha was 0.80. Workplace support was measured using an eight-item scale that included co-workers’ and supervisors’ instrumental and socio-emotional support. Ex.: “My co-workers are willing to listen to my problems.” Cronbach’s alpha was 0.86 for co-workers’ support and 0.91 for supervisors’ support.

#### Work Engagement

The Utrecht Work Engagement Scale (UWES-17) (Schaufeli and Bakker, 2003, Unpublished) was applied in our study as a single-factor construct measured using 17 items measured on a seven-point Likert scale ranging from 0 (never) to 6 (always). In Slovene was translated by Zager Kocjan (2016). It included vigor, dedication and absorption. The *vigor* category includes six items (ex.: “I feel strong and full of energy at work.”), the *dedication* category includes five (ex.: “I believe that the work I do has sense and purpose.”), and the *absorption* category includes six (ex.: “When I am at work, I forget about everything around me.”). Cronbach’s alpha was 0.95.

#### Corporate Volunteering Climate

The *corporate volunteering climate* was measured using a scale for measuring the corporate volunteering climate (Rodell et al., 2017). We used the established systematic approach of a familiarity- and recognizability-driven adaptation of a questionnaire (Malda et al., 2008) when translating it to Slovene. The translation process was done according to expert recommendations (e.g., Hambleton and De Jong, 2003) and thus included translation and back translation. The scale included five items measured on a five-point Likert scale ranging from 1 (almost never) to 5 (very often). Employees responded to matter-of-fact statements such as “Employees at my organization participate in volunteering activities.” Cronbach’s alpha was 0.95.

#### Corporate Volunteering

A yes or no question was asked whether the employees participate in their organizations’ volunteering activities and whether the companies implement corporate volunteering programs.

#### Control Variables

Gender, age and education were included as control variables in regression models.

### Procedure

We contacted the organizations based on data indicating that their employees participate in volunteer activities. Slovenska Filantropija (Slovenian Philanthropy), a non-profit that works with companies to promote volunteering, also participated in the sampling. We sent invitations to participate in the survey to the e-mail addresses of the companies’ directors or heads of HR. We presented the purpose and the design of the study, how their data would be used, ethical consideration and on the value of the research for their organizations.

To avoid the reliability problems with question of single-item measures (Kenny, 1979; Hinkin, 1995) we checked the involvement in volunteer programs for each participant twice – based on his/her response on the single-item question *Do you participate in your organizations’ volunteering activities?* and on HR manager’s or director’s answer on *Is your organization involved in volunteering?* There were four possible combinations of answers to those questions:

- Yes, I participate + Yes, my organization is involved = yes
- Yes, I participate + No, my organization is not involved = yes (31% answers. We checked the background of these participants, and they all work in educational sector (schools), where volunteering is an essential activity included on weekly basis of their programs (e.g., collecting old paper, collecting old clothes, helping vulnerable groups of pupils…). So, these participants do volunteer activities at their workplace.
- No, I do not participate + Yes, my organization is involved = yes (6.2% answers; based or research by Rodell et al. (2017) these participants can perceive the corporate volunteering climate, without having to actively participate in volunteering programs. This was concluded from the answers of the employees of the same company. They all answered their organization implements volunteering activities, but some answered they actively participate, while others don’t.)
- No, I do not participate + No, my organization is not involved = no

The online self-reported survey battery was sent to all of the employees of the individual organizations, while their participation was anonymous and voluntary. The survey battery was administered in line with the Slovene Law (Personal Data Protection Act 2004-01-3836 and subsequent amendments) and the ethical standards for research approved by the Ethical Committee at the Faculty of Arts, University of Ljubljana (Slovenia). The study and protocol were reviewed and approved by the Ethical Committee at the Faculty of Arts, University of Ljubljana (Slovenia). The consent of the participants was obtained by virtue of survey completion. The participants were told also that they could withdraw from the study at any time and that there would be no payment for participating.

The data was collected in the spring of 2018, and was analyzed using the computer program SPSS.

## Results

The first step of the analysis was testing validity of the used measurement scales. A multilevel confirmatory factor analysis was run for this purpose. The measurement model was complex and the sample size at the company level was relatively low, therefore a model within and between groups was constrained to equal measurement errors, covariances and variances. Only indicator loadings were estimated freely. The fit indices are within the specifications (Hair et al., 2006), with a RMSEA of 0.077 (specification <0.08). Due to the large sample size the chi square test was statistically significant (χ^2^ = 2814.7; *p* < 0.001), but the χ^2^/df ratio at a value of 1.87 indicated a good model fit (specification < 2.0 or < 3.0). The validity of the model was also tested using a pooled-within cluster covariance matrix as suggested by Hox (2002). In this instance the analysis explained variation within organizations taking into account the nested nature of the data. The analysis was therefore performed only at the level of employees, where the sample size is sufficiently large. The fit indices suggest an acceptable fit of the model as follows: RMSEA = 0.07, χ^2^ = 1606.1 (*p* < 0.001), χ^2^/df = 2.2, SRMR = 0.07. SRMR values as high as 0.08 are deemed acceptable (Hu and Bentler, 1999).

After establishing adequate measurement validity and reliability (Cronbach’s α > 0.70 for all scales), composite (mean) scores for all scales were calculated. Means, standard deviations and correlations for respondents’ age, gender and composite variables are summarized in **Table 1**. Corporate volunteering is positively and statistically significantly associated with the corporate volunteering climate, work engagement, autonomy, co-workers, and supervisors support. Items measuring variety are reverse coded and higher values mean higher monotony of work. This explains the mainly negative associations with other variables.

**Table 1:** Descriptive statistics and correlations between factors.

	M	SD	Male	Age	HE	VC	CVC	WE	V	A	CS	SS	PWD
Male	0.43	0.50	1										
Age	40.9	9.76	-0.02	1									
HE	0.52	0.50	-0.20^**^	0.10	1								
VC	0.62	0.49	-0.08	0.05	0.09	1							
CVC	3.08	0.91	-0.18^**^	0.13^*^	0.16^**^	0.30^**^	1						
WE	5.20	0.99	-0.01	0.23^**^	0.17^**^	0.22^**^	0.32^**^	1					
V	1.72	0.72	-0.06	-0.21^**^	-0.12	0.02	-0.14^*^	-0.51^**^	1				
A	3.59	0.84	0.04	-0.02	0.16^**^	0.19^**^	0.21^**^	0.45^**^	-0.41^**^	1			
CS	3.83	0.83	-0.04	-0.09	0.01	0.24^**^	0.22^**^	0.26^**^	-0.05	0.23^**^	1		
SS	3.51	1.05	-0.14	-0.10	0.10	0.21^**^	0.22^**^	0.37^**^	-0.06	0.29^**^	0.54^**^	1	
PWD	4.10	0.67	0.06	0.21^**^	-0.01	-0.09	0.00	0.26^**^	-0.45^**^	0.15^**^	-0.07	-0.12^*^	

*CVC = corporate volunteering climate; WE = work engagement; PWD = perceived work demand; SS = supervisor support; CS = co-worker support; A = autonomy; V = variety; HE = high education, second level university education or more; VC = corporate volunteering. ^∗^p < 0.05; ^∗∗^p < 0.01.*

In order to test the hypothesis about the relationship between the participation in corporate volunteering and the corporate volunteering climate, we employed a mixed linear regression analysis with random intercept (**Table 2**). The fixed effects in the model were gender, age, education, and corporate volunteering. The dependent variable was the corporate volunteering climate. The results suggest there is a statistically significant and positive relationship between the participation in corporate volunteering and the perceived corporate volunteering climate when controlling for gender, age, and education of respondents.

**Table 2:** Influence of corporate volunteering on the perception of the corporate volunteering climate (results of multiple linear mixed regression).

	Est. (*p*-value)
**Fixed eff.**	
Intercept	2.19 (<0.001)
Female	0.1 (0.407)
Age	0.01 (0.092)
Higher education	0.16 (0.15)
VC	0.63 (<0.001)
**Random eff.**	Est. (95% CI)
Intercept	0.10 (0.03-0.36)

*High education = second level university education or more; VC = corporate volunteering.*

To test the hypothesis about the relationship between corporate volunteering and job characteristics, a mixed linear regression analysis with random intercept was performed. The fixed effects in the model were the same as in the previous analysis. Several regression models were built with each job characteristic as a dependent variable (**Table 3**). When controlling for gender, age and education, corporate volunteering is positively and statistically significantly associated with autonomy and co-worker support. The differences in the reporting of the variability of work duties and supervisor support did not appear as statistically significant and therefore we only partially confirmed our hypothesis.

**Table 3:** Influence of corporate volunteering on job characteristics (results of multiple linear mixed regression).

	Variety	Autonomy	Co-worker support	Supervisor support	Perceived work demand
	
	Est. (*p*-value)	Est. (*p*-value)	Est. (*p*-value)	Est. (*p*-value)	Est. (*p*-value)
**Fixed eff.**					
Intercept	2.45 (<0.001)	3.32 (<0.001)	3.86 (<0.001)	3.68 (<0.001)	3.46 (<0.001)
Female	0.09 (0.397)	-0.16 (0.18)	-0.02 (0.89)	0.11 (0.463)	-0.04 (0.652)
Age	-0.02 (<0.001)	0 (0.973)	0 (0.556)	-0.01 (0.169)	0.02 (<0.001)
High education	-0.2 (0.025)	0.34 (<0.001)	-0.06 (0.567)	0.12 (0.362)	0.04 (0.619)
VC	-0.05 (0.624)	0.33 (0.003)	0.28 (0.011)	0.25 (0.075)	-0.08 (0.391)
**Random eff.**	**Est. (95% CI)**	**Est. (95% CI)**	**Est. (95% CI)**	**Est. (95% CI)**	**Est. (95% CI)**
Intercept	0.07 (0.02–0.22)	010 (0.03–0.30)	0.11 (0.03–0.34)	0.13 (0.04–0.45)	0.04 (0.01–0.15)

*High education = second level university education or more; VC = corporate volunteering.*

A multilevel linear regression model with random intercept was built to test the hypothesis that corporate volunteering is associated with work engagement (**Table 4**). When controlling for gender, age and education, corporate volunteering is positively and statistically significantly associated with work engagement. Participants whose employers implement corporate volunteering programs are more enthusiastic about their work and tend to work with higher vigor, dedication, and absorption.

**Table 4:** Influence of corporate volunteering on work engagement (results of multiple linear mixed regression).

	Work engagement
	Est. (*p*-value)
**Fixed eff.**	
Intercept	4.08 (<0.001)
Female	-0.2 (0.131)
Age	0.02 (<0.001)
High education	0.26 (0.026)
VC	0.4 (0.002)
**Random eff.**	Est. (95% CI)
Intercept	0.15 (0.05–0.45)

*High education = second level university education or more; VC = corporate volunteering.*

## Discussion

After the findings that corporate volunteering offers numerous benefits to employees, employers and the community at large, increasing numbers of companies began to introduce their own volunteer programs, through which they motivate their employees to participate in their corporate volunteering activities. On the other hand, young job seekers increasingly report corporate social responsibility as an important factor of the employer’s brand. In this study we wished to analyze the role of corporate volunteering programs in the perception of job resources and in the development of employee engagement. We are first who, following Rodell et al. (2017), measured the corporate volunteering climate at companies regardless of whether their employees actively participated in corporate volunteering or not. The research in question (Rodell et al., 2017) showed that the corporate volunteering climate influences employees’ affective commitment to their employer and increases the motivation of non-volunteer employees to participate in volunteering activities. In view of the fact that the corporate volunteering climate can be developed both by employees who express interest in volunteer activities and through volunteer programs initiated by the companies themselves (Rodell et al., 2017), we were able to measure it at the level of each individual and also observe its relationship to incentives from the work environment (e.g., autonomy, supervisor support).

The results showed that actively participating in volunteer activities significantly correlates with employees’ perception of the corporate volunteering climate at their company. Thus we were able to confirm the first hypothesis, as it was shown that employees who do not actively participate in volunteer activities perceive the corporate volunteering climate to a significantly lesser degree than those who do actively participate in volunteer activities. This finding does not agree with the results of Rodell et al. (2017), that when a corporate volunteering climate is created, individual employees don’t necessarily have to participate in volunteer activities to perceive it. However, the corporate volunteering climate can lead to non-volunteers becoming more willing to participate in various volunteer activities offered by their employer (Rodell et al., 2017), which could consequently raise the corporate volunteering climate among those who currently do not actively participate in volunteer activities.

We tested the connection between corporate volunteering and work engagement and job resources using two hypotheses. We assumed that employees who are employed in organizations that implement the corporate volunteering programs would report higher levels of job resources in their work environment than those whose employers don’t carry out volunteering programs. The results showed that carrying out a volunteering program within an organization positively correlates with the perception of certain job resources. Those whose employers implement volunteering programs report more autonomy and support from their co-workers at the workplace than those whose employers do not implement such activities. This corresponds with past findings, as employees who participate in corporate volunteering have more contacts with others, both their own co-workers and with other participants in the volunteer activities, which gives them a sense of belonging (cf. Brockner et al., 2014), allows them to develop interpersonal skills, communication and active listening (Booth et al., 2009), and to experience more positive emotions than negative ones (Mojza et al., 2011). All of this can lead to the easier development of positive attitudes at the workplace, which results in an increased perception of support from co-workers. Due to the time that employees have to dedicate to volunteering while at work, they also require a certain amount of autonomy, both in planning and organizing and also in decision-making, which consequently gives them more opportunities to learn new techniques and leadership and management skills (Booth et al., 2009) and thus also leads to better job performance (Rodell et al., 2016). Since volunteer activities at the workplace constitute an additional task (i.e., workload) that requires the use of various skills, employees might not try to increase the level of diversity of their basic employment duties, which is also supported by our research.

We were able to confirm the hypothesis that corporate volunteering correlates positively with work engagement, as employees whose employers carry out volunteer activities are more engaged in comparison with employees whose employers do not. Thus implementing volunteer activities gives employees more energy and stamina for doing their work, makes them more engaged in their work, and gives them more of a sense of the meaningfulness and importance of their work. These results are consistent with Allen’s (2013) finding that the indirect satisfaction of employees’ needs through volunteering could serve as a basis for a positive correlation between these two constructs. However, some authors (e.g., Caudron, 1994; Booth et al., 2009; Breitsohl and Ehrig, 2016; Rodell et al., 2016, 2017) have reported a positive effect of corporate volunteering on other organizational behaviors (productivity, job satisfaction, sense of belonging, performance), which are positively correlated with work engagement (Harter et al., 2013; Karanika-Murray et al., 2014; Kim et al., 2017).

### Implications for Practice

Companies employ several strategies in order to be more socially responsible. One of them, which is attracting increasing interest and which is being employed increasingly frequently around the world, is corporate volunteering, i.e., the planning and implementation of formal volunteer programs and participation in volunteer activities within the organization. In view of the fact that this activity is becoming increasingly popular and that it has the ability to affect several significant social issues, it is important to understand how it can influence the viewpoints and behavior of employees whose employers carry out corporate volunteering programs.

The research results provide useful findings not only in social but also in organizational psychology. The results of the current study could also motivate employers who are not engaged in corporate volunteering to develop their own volunteering programs, through which they could provide additional motivation for a larger number of employees. Although there are still some open questions regarding the added value of corporate volunteering, in the future it could become one of the key activities of corporate social responsibility through which companies with adequate (employee-friendly) volunteer programs could improve various job resources, work engagement, the employees’ sense of belonging and finally also their performance.

Our study indicates that companies require better communication about volunteer activities – both about their added value for each individual and for all employees, particularly with regard to programs that companies are already operating, but which non-volunteers either do not know about or do not recognize their benefits.

#### Limits to the Study and Guidelines for Further Research

When interpreting the results of our research, it is important to understand its limitations, particularly with regard to the data collection process. First, the respondents filled out online questionnaires, meaning that only people with internet access at work could participate. Second, since the questionnaires were first sent to directors and the heads of HR departments, the number of completed questionnaires could also be dependent on the methods used to motivate their employees to complete them. Third, when communicating with the management of certain companies we noticed that some people were not familiar with the concept of corporate volunteering, even though they had already participated in various volunteer activities in cooperation with Slovenska Filantropija. Therefore we recommend that in future research the participants should be familiarized with the term corporate volunteering in advance, since it is a fairly new phenomenon. Fourth, the single item scale was used to evaluate the nature of the corporate volunteering participation. Information about the duration, specificity, level of inclusion to the specific volunteering program are missing in our study.

Future research will have to include accurate data on the forms of corporate volunteering (i.e., whether participation is mandatory or non-mandatory, during or outside of work time, its frequency and other circumstances relating to volunteer activities). The question of the role of corporate volunteering in the development of the employer’s brand, i.e., what affect such an organizational culture has on job seekers, customers or investors, remains open, since organizations that engage in volunteer activities are allegedly more attractive on the job market (cf. Booth et al., 2009; Rodell et al., 2016). However, it will clearly be necessary to shift from the individual to the corporate level, at which it will be possible to compare the organizations that conduct volunteer programs with those that do not.

## Conclusion

Our study makes several contributions. At the theoretical level we presented the connections between various individual factors that can affect the development of the corporate volunteering climate, and discussed the transfer of findings to the organizational level. We used a questionnaire for measuring the corporate volunteering climate, which served as a concise, practical and reliable tool. We confirmed that the volunteer activities organized or conducted by employers correlates positively with engagement and the perception of job resources (autonomy and support from co-workers), which is undoubtedly significant for both the company as a whole and for its management. We observed that in order to improve the perception of the corporate volunteering climate it is necessary to invite all employees within an organization to actively participate. This topic has therefore revealed several new research challenges, which will be surmountable only with the support of employers, through the continued introduction, development, implementation, and valuation of volunteer activities in the work environment.

## Author Contributions

All authors were involved in the conceptualization of the research problem, research design, interpretation of data, and the final writing process.

## Conflict of Interest Statement

The authors declare that the research was conducted in the absence of any commercial or financial relationships that could be construed as a potential conflict of interest.
